# Supplementary material for: Cognitive Function, Healthy Lifestyle, and All-Cause Mortality among Chinese Older Adults: A Longitudinal Prospective Study
Source: Nutrients. 2024 Apr 26;16(9):1297. doi: 10.3390/nu16091297 (PMC11085585; doi:10.3390/nu16091297)
Supplement: Supplementary file 1 [file nutrients-16-01297-s001.zip › nutrients-2964434-supplementary.pdf]

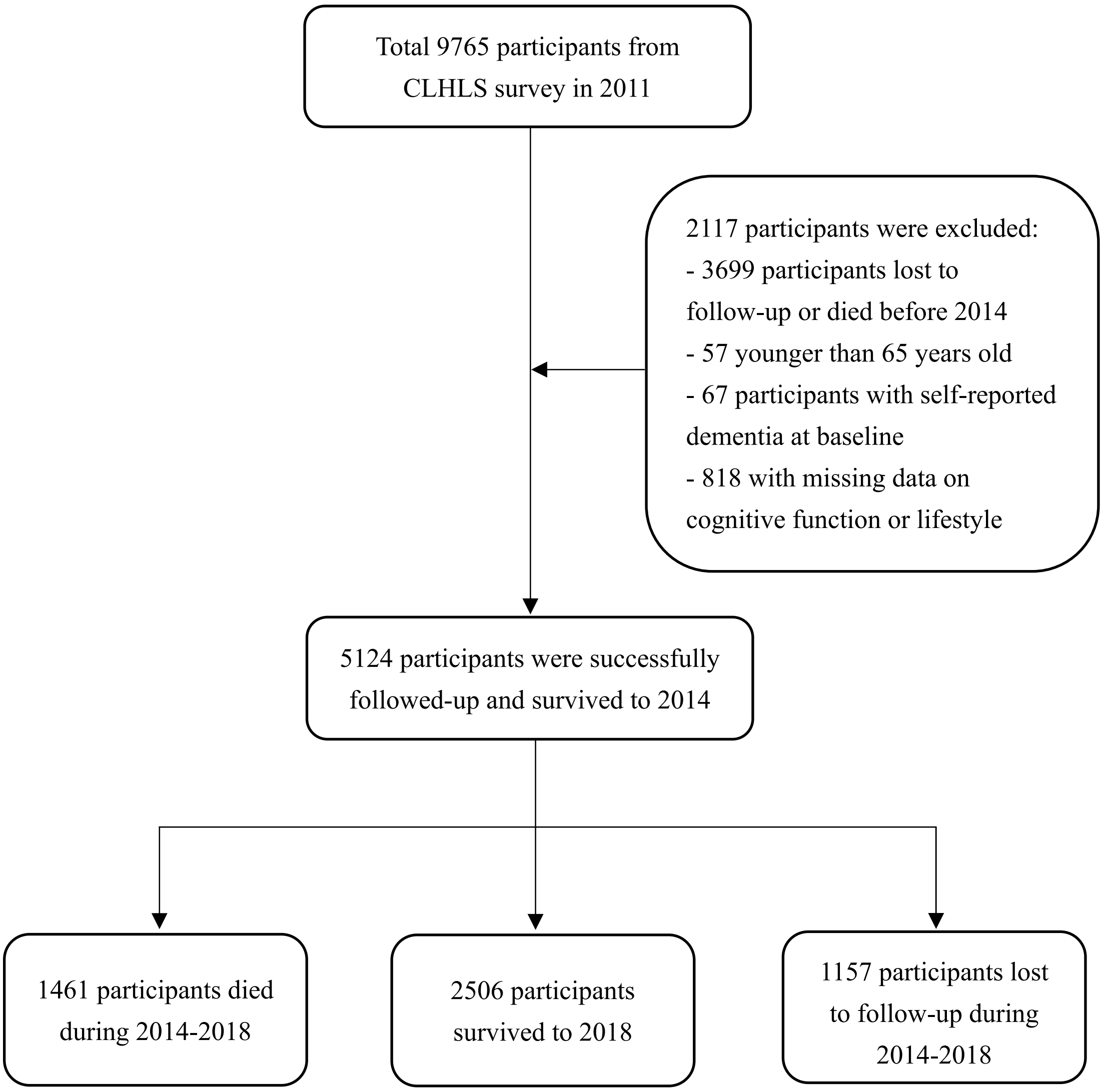

Figure S1. Flow chart of the included CLHLS participants

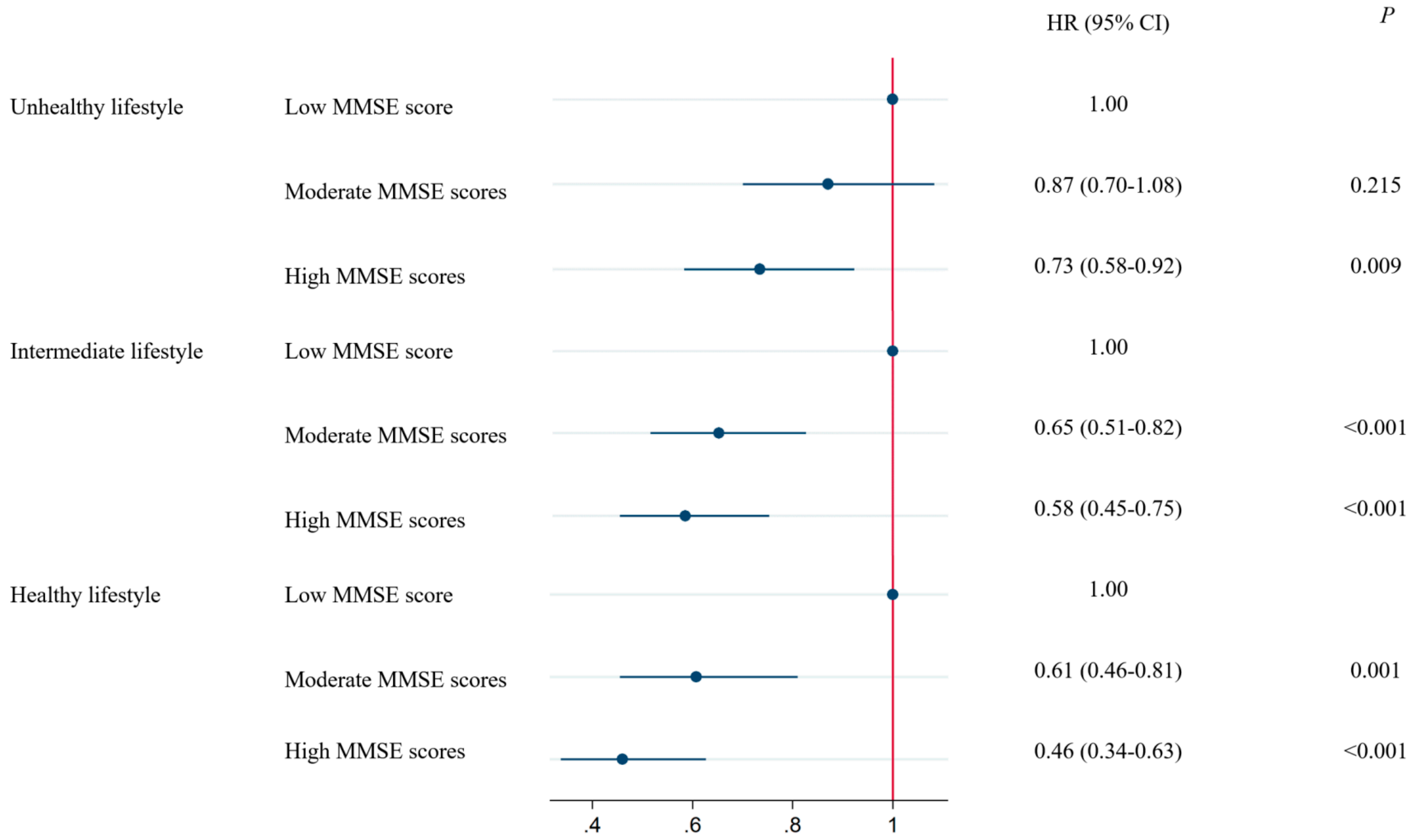

Figure S2. Associations of the MMSE scores with mortality stratified by lifestyles. Results were adjusted for age, sex, education, residence, marital status, living pattern, self-rated of economic status, ADL in disability and history of chronic disease (diabetes, heart diseases, cancer and stroke). MMSE, Mini-Mental State Examination; HR, hazard ratio; CI, confidence interval.
